# A Simplified Computational Strategy Focused on Resin Damage to Study Matrix Cracking of The Cross-Ply Laminates Under Uniaxial Tension Load

**DOI:** 10.3390/ma12121984

**Published:** 2019-06-20

**Authors:** Liangliang Sun, Jihui Wang, Haixiao Hu, Aiqing Ni

**Affiliations:** 1 School of Material Science and Engineering, Wuhan University of Technology, 122 Luoshi Road, Wuhan 430070, China; sunliangliang@whut.edu.cn (L.S.); jhwang@whut.edu.cn (J.W.); hxhu@whut.edu.cn (H.H.); 2 State Key Laboratory of Advanced Technology for Materials Synthesis and Processing, Wuhan University of Technology, 122 Luoshi Road, Wuhan 430070, China

**Keywords:** transverse cracking, multi-scale modeling, finite element analysis, damage mechanics, residual stress

## Abstract

Transverse cracking is probably the first and most dominant mode of damage in composite materials. In this paper, transverse cracking of cross-ply [0_2_/90_n_]_s_ (n = 2,3,4) laminates under uniaxial tension load was studied by means of experimental and numerical methods. In the numerical simulations, a simplified computational strategy only focusing on the damage of the resin was proposed and the mechanical response of the cracking cross-ply laminates was studied by finite element analysis of multi-scale representative volume elements (RVEs). In the RVEs, the longitudinal 0° plies were represented by macro-scale, homogeneous, orthotropic elastic solids while the 90° plies were modeled by the discrete fibers and the surrounding matrix resin in micro-scale. Based on researching the critical longitudinal mechanical strain $\varepsilon_{x}$ which initiates the cracks, the in-situ transverse ply strength and the stiffness degradation of the transverse plies, the simplified computational strategy proposed was proven correct. In addition, the crack initiation is sensitive to residual stress. Higher process-induced residual stress levels are dangerous to laminates, leading to early crack initiation.

## 1. Introduction

Fiber reinforced polymers (FRP) are extensively used in modern engineering applications which require high mechanical properties and safe reliability. Therefore damage initiation and accumulation in FRP are essential for the design, production and health monitoring of composite structures. Nucleation and propagation of transverse matrix cracking do not normally lead to structural collapse very quickly but degrade the damage resistance and lead to other damage modes like delamination. There is a strong interaction and coupling between transverse matrix cracking and delamination. In general, micro transverse cracks initiate first, combine and propagate until they extend to the ply boundaries, at which point local micro-delamination is triggered. Then, delamination becomes the dominant failure mechanism after the transverse crack density has reached saturation up to the catastrophic failure of the composite structure [1]. In a sense, the onset of transverse matrix cracking is the origin of damage for composite structures. Therefore, where and when the transverse matrix cracking initiates and how it propagates are of great interest to the researchers focused on composite damage.

The earliest and simplest modeling technique to address matrix damage is perhaps the ply discount method [2]. The mechanical response of matrix cracking and delamination is now well established by damage mechanics and fracture mechanics [3,4,5,6,7,8]. In the literature, many efforts have been devoted to the relationship of the laminate elastic properties to the matrix cracks. The most common of them could be classified as: finite element (FE) analysis of cracked laminates [9,10], crack faces displacement (CFD) or crack opening displacement (COD) models [11,12], synergistic damage mechanics (SDM) models [13,14], variational analysis [15,16], generalized plane strain analysis (McCartney’s models) [17] and shear lag (SL) analysis [18,19]. In most of the research, the laminates were modeled in the presence of regular crack arrays which means the cracks were pre-located or pre-existing. In fact, composites have complex micro structures in nature. As the research moves along, more details about the matrix damage are paid attention to. With the help of microscopic models and computational mechanics, problems closer to the physical nature of matrix damage could be solved. Micro-scale models and multi-scale models describing the properties of composite materials have been sprung up recent years. In these models, the information about fibers, resin, interface, weaving details of the fabric and various kinds of defects were included [20,21,22,23,24]. In this paper, we aim to create a model to study the details about when a crack initiates and how it propagates without pre-cracks and the correlation of the laminate’s elastic properties to the cracks.

It has been reported that transverse matrix cracks can be initiated by fiber-resin interface decohesion [25,26]. In numerical simulations, decohesion behavior is normally described by the cohesive method or virtual crack closure technique (VCCT). VCCT was not considered as it needs pre-cracks. The cohesive constitutive relationships and cohesive elements themselves determine that severe convergence problems usually occur, especially in an implicit algorithm, which leads to the early termination of the program. Both literatures [25,26] encountered such problems. Thus the cohesive method was also given up. In this paper, we aim to establish a simple method from a damage mechanics perspective to characterize the mechanical behaviors of cracking in cross-ply laminates under uniaxial tension load. A new damage degradation method for the special purpose of resin damage is proposed here. In the computational strategy, any cohesive behaviors including the interface decohesion between fibers and resin and the delamination between the ply boundaries were ignored. Only the damage of the resin matrix was taken into account. In this way, the numerical computation is much easier to converge. The commercial implicit analysis program ABAQUS/Standard was used to help finish the analysis. Now the only concern is whether this kind of simplified method can catch the internal characteristics of matrix cracking. Therefore, the experiment of observing the transverse cracks at the cross section of the transverse plies and some data from the literature were implemented to validate the computational strategy.

## 2. Experiment

### 2.1. SEM Tests of the Cross Section of the Transverse Plies

The cross-ply laminates were produced by Hexply AS4/8552 prepreg in an autoclave under the recommended processing by Hexcel. After the curing process, the laminate plates were demoulded and cut into samples with specific shapes in preparation for testing. The specimens for Scanning Electron Microscope (SEM, JSM-IT300, JEOL, Tokyo, Japan) tests were cut perpendicular to the fiber alignment direction to expose the desired cross section by diamond disk. Then polishing was carried out by sandpaper of 400, 800, 1500, 3000 and 7000 mesh, successively, to obtain a flat surface with a mirror-like finish. At last the polished surfaces of the specimens were tested by a SEM machine. The fiber volume fraction and the radius of the carbon fibers were determined from the photographs of SEM tests. 

### 2.2. Uniaxial Tensile Test to Observe the Transverse Cracks

**Figure 1:**
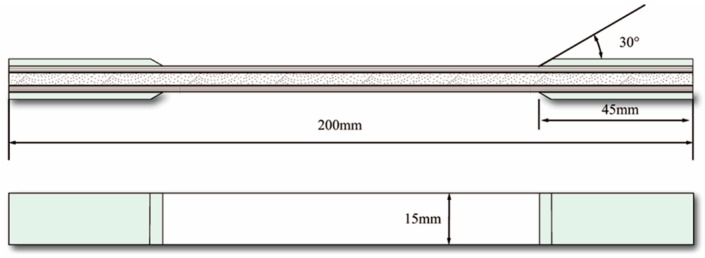
The configuration and the dimension of the specimens for the uniaxial tensile test.

**Figure 2:**
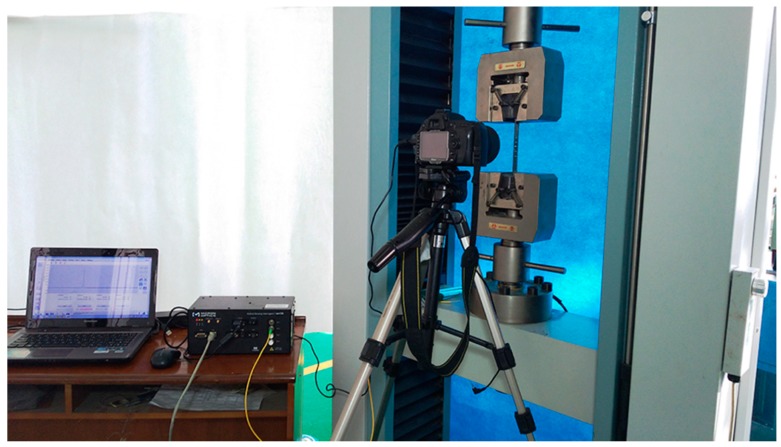
Photograph of the testing system for the transverse crack test.

The uniaxial tensile test referred to standards of American Society for Testing Materials (ASTM) D638 and D3039. The specimens for the tensile test were cut into a strip of 200 mm long and 15 mm wide and E-glass/epoxy stiffener tabs were attached on the surfaces of each end (see Figure 1). The tensile test was performed using the universal testing machine (CIMACH, DNS100, Changchun Research Institute for Mechanical Science Co., Ltd, Changchun, China) with a 100 kN load cell by a displacement rate of the crosshead at 0.5 mm/min. Here a Nikon D90 camera (Nikon, Tokyo, Japan) and a 65 mm macro lens (Nikon, Tokyo, Japan) was used to observe the transverse cracks in the cross section of the specimen. With the help of a computer, the images were captured every 5 s automatically. The testing system is shown in Figure 2. The number of the cracks within the marked 2 cm long edge of the cross section was counted through the loading history from the captured photos. Crack density is defined as the number of cracks per unit length normal to the crack faces. Therefore the crack density was then calculated by using the counted crack numbers divided by 2 cm. More details can be found in the previous work [27].

## 3. Computational Methodology

**Figure 3:**
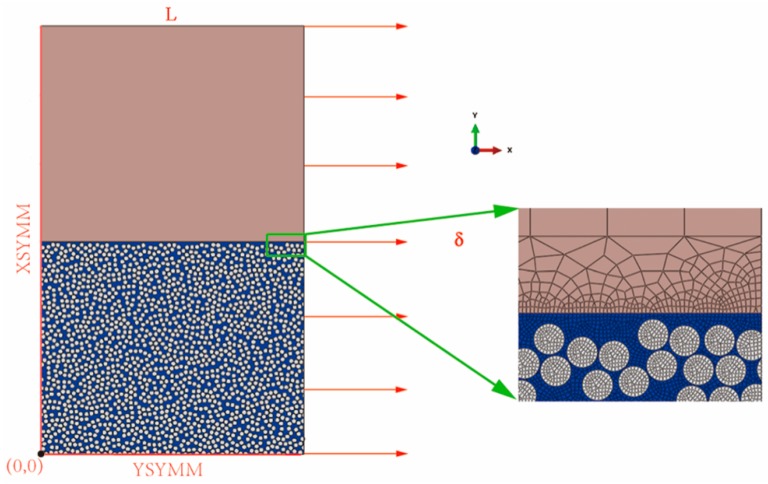
The finite element representative volume element (RVE) model of the [0_2_/90_2_]_s_ laminate with the load and boundary conditions applied and details of the finite element mesh.

A multi-scale modeling strategy was used to model transverse matrix cracking in cross-ply [0_2_/90_n_]_s_ (n = 2,3,4) laminates in order to get a high fidelity model. The normal thickness for a single ply is 0.18 mm [27] and the length of the representative volume element (RVE) model was 1 mm so an adequate number of fibers could be modeled to ensure an appropriate crack growth. The finite element model is shown in Figure 3. In the RVE model, 0° plies were modeled in macroscopic scale and the 90° plies were modeled in microscopic scale. It should be indicated that this is a 2D model and only the through thickness cracks can be accounted for. We assumed that the laminate would not deform and the load was uniform along the width direction. Thus, the laminates were under plane strain state and the discussions below are all under this condition. 

The 0° plies of the cross-ply laminates were assumed to behave as homogenized, transversely isotropic solids with elastic constants in Table 1 [26]. While in the 90° plies, the carbon fibers were modeled as transversely isotropic solids, with the elastic modulus of 13 GPa and the Poisson’s ratio of 0.46. The thermal expansion of the AS4 fiber was 7.2 × 10^−6^ /K [26]. The fiber radius *R* was assumed to be constant and it was an experimental value. In order to achieve the target fiber volume fraction, the modified random sequential adsorption algorithm was adopted [28]. It should be noted that additional constraints like the minimum distance between fiber surfaces (>0.07 *R*) and between the fiber surface and the ply edges (>0.1 *R*) were added to the algorithm to get a reasonable finite meshing. 

The rest area of the 90° plies was modeled as the resin. The epoxy resin was simply assumed to be isotropic elastic-brittle materials. Normally, a scalar damage variable is suitable for the damage of isotropic materials. Nevertheless, the elastic property of a damaged material due to distributed microcracks or high volume fraction of inclusions could be treated as orthotropic [29]. Therefore, we use a damage tensor of second-order to describe the damage of the resin. In continuum damage mechanics, damage effect tensor M is used to specify the effective stress $\hat{\sigma }$ and the nominal stress σ:

(1) $$\hat{\sigma }=M\sigma$$

and M has a diagonal form:

(2) $$M=\left[\begin{array}{ccc} \frac{1}{1-d_{mI1}} & 0 & 0\\ 0 & \frac{1}{1-d_{mI2}} & 0\\ 0 & 0 & \frac{1}{1-d_{s}} \end{array}\right];\;I\in \left\{t,c\right\}$$

where $d_{mI1}$ and $d_{mI2}$ are damage variables according to the damage in two vertical directions in Cartesian coordinate; I is the failure mode; the subscripts t or c represent tension or compression damage, respectively; $d_{s}$ is a damage variable for shear failure mode. Here, it is related to $d_{mI1}$ and $d_{mI2}$:

(3) $$d_{s}=1-(1-d_{mI1})(1-d_{mI2})$$

According to Ireneusz and Juan [30] and Laws et al. [31], the damaged compliance matrix can be written as:

(4) $$S_{d}=\left[\begin{array}{ccc} \frac{1-\nu^{2}}{(1-d_{mI1})E} & -\frac{\nu (1+\nu )}{E} & 0\\ -\frac{\nu (1+\nu )}{E} & \frac{1-\nu^{2}}{(1-d_{mI2})E} & 0\\ 0 & 0 & \frac{2(1+\nu )}{(1-d_{s})E} \end{array}\right]$$

where E and ν are the undamaged elastic modulus and Poisson’s radio for the resin matrix, and the corresponding damaged stiffness matrix is:

(5) $$C_{d}=\frac{1}{D}\left[\begin{array}{ccc} \frac{(1-d_{mI1})(1-\nu )E}{\left(1+\nu \right)} & \frac{(1-d_{mI1})(1-d_{mI2})\nu E}{\left(1+\nu \right)} & 0\\ \frac{(1-d_{mI1})(1-d_{mI2})\nu E}{\left(1+\nu \right)} & \frac{(1-d_{mI2})(1-\nu )E}{\left(1+\nu \right)} & 0\\ 0 & 0 & D(1-d_{s})\frac{E}{2(1+\nu )} \end{array}\right]$$

where $D={\left(1-\nu \right)}^{2}-\nu^{2}(1-d_{mI1})(1-d_{mI2})$.

**Figure 4:**
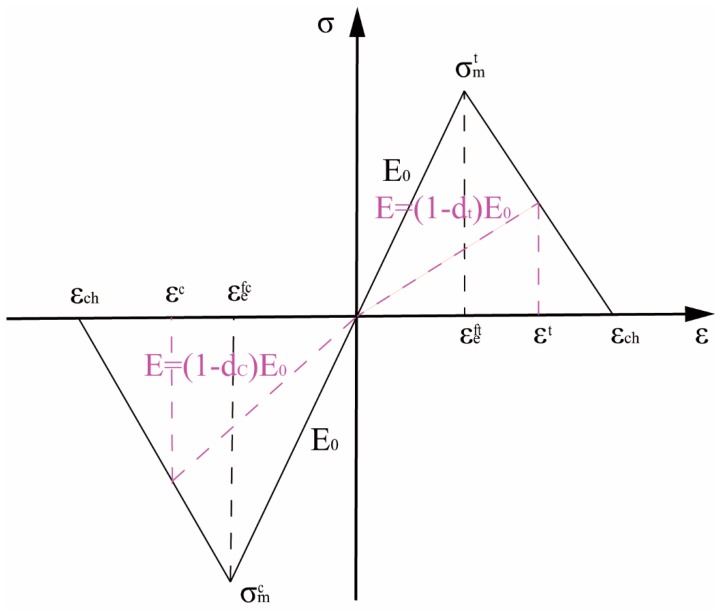
The schematic of damage initiation and evolution of the uniaxial tension-compression response for the epoxy resin.

The schematic of damage initiation and evolution of the resin is shown in Figure 4. At the beginning, the material is linear elastic till the tensile strength $\sigma_{m}^{t}$ or compressive strength $\sigma_{m}^{c}$ is reached. In the post-peak regime, the material shows linear softening behavior. Although in reality, the epoxy resin exhibits plastic behaviors, especially in compression, it is assumed to be a brittle material here to simplify the computational process. The post-peak behavior is controlled by the fracture energy $G_{m}$ dissipated in the damage process. The mechanical properties of the resin are listed in Table 2 [15].

Enlightened by Hashin’s criteria for matrix damage [32], damage initiation refers to the onset of materials’ degradation and the initiation criteria were expressed as:

Tension in 1-drection (${\hat{\sigma }}_{11}\geq 0$):

(6) $$F_{1t}={\left(\frac{{\hat{\sigma }}_{11}}{\sigma_{m}^{t}}\right)}^{2}+{\left(\frac{{\hat{\sigma }}_{12}}{\sigma_{m}^{s}}\right)}^{2}=1$$

Compression in 1-drection (${\hat{\sigma }}_{11}<0$):

(7) $$F_{1c}={\left(\frac{{\hat{\sigma }}_{11}}{2\sigma_{m}^{s}}\right)}^{2}+\left[{\left(\frac{\sigma_{m}^{c}}{2\sigma_{m}^{s}}\right)}^{2}-1\right]\frac{{\hat{\sigma }}_{11}}{\sigma_{m}^{c}}+{\left(\frac{{\hat{\sigma }}_{12}}{\sigma_{m}^{s}}\right)}^{2}=1$$

Tension in 2-drection (${\hat{\sigma }}_{22}\geq 0$):

(8) $$F_{2t}={\left(\frac{{\hat{\sigma }}_{22}}{{\hat{\sigma }}_{m}^{c}}\right)}^{2}+{\left(\frac{{\hat{\sigma }}_{12}}{{\hat{\sigma }}_{m}^{s}}\right)}^{2}=1$$

Compression in 2-drection (${\hat{\sigma }}_{22}<0$):

(9) $$F_{2c}={\left(\frac{{\hat{\sigma }}_{22}}{2\sigma_{m}^{s}}\right)}^{2}+\left[{\left(\frac{\sigma_{m}^{c}}{2\sigma_{m}^{s}}\right)}^{2}-1\right]\frac{{\hat{\sigma }}_{22}}{\sigma_{m}^{c}}+{\left(\frac{{\hat{\sigma }}_{12}}{\sigma_{m}^{s}}\right)}^{2}=1$$

where ${\hat{\sigma }}_{ij}$ refers to components of effective stress tensor; $\sigma_{m}^{t}$, $\sigma_{m}^{c}$ and $\sigma_{m}^{s}$ are tensile strength, compressive strength and shear strength of the resin matrix, respectively. 

It is well-known that in continuum mechanics, the constitutive model is normally expressed as stress–strain related equations. This formulation results in a serious mesh dependency of the numerical results of strain-softening materials because of strain localization and the energy dissipated decreases with mesh refinement. Mesh regularization can be achieved by the crack band model [33], in which the limit strain $\varepsilon^{f}$ is expressed as:

(10) $$\varepsilon^{f}=\frac{2G_{m}}{\sigma_{m}^{I}L_{c}}$$

where $L_{c}$ is the characteristic length of the element. If the softening is linear, Equation (10) can be transformed into:

(11) $$\delta_{I,eq}^{f}=\frac{2G_{m}}{\sigma_{I,eq}^{0}}$$

where $\delta_{I,eq}^{f}$ is the equivalent displacement at which the material is fully damaged and $\sigma_{I,eq}^{0}$ is the equivalent stress at which the damage initiation criterion is satisfied. In this way, the stress–strain relationships could be changed into stress–displacement relationships. The damage variable for each failure mode is given as:

(12) $$d_{mI}=\frac{\delta_{I,eq}^{f}\left(\delta_{I,eq}-\delta_{I,eq}^{0}\right)}{\delta_{I,eq}\left(\delta_{I,eq}^{f}-\delta_{I,eq}^{0}\right)}\begin{array}{ccc} ; & & \delta_{I,eq}^{0}\leq \delta_{I,eq} \end{array}\leq \delta_{I,eq}^{f}$$

where $\delta_{I,eq}^{0}$ and $\delta_{I,eq}$ are equivalent displacement at which the damage initiation criterion is satisfied and at current state. Thus, the damage evolution is governed by the equivalent displacement. The equivalent displacement and equivalent stress definitions are listed in Table 3. In the damage initiation, the equivalent displacement and stress are computed by multiplying a scaling factor:

(13) $$\begin{array}{l} \delta_{I,eq}^{0}=\delta_{I,eq}f^{sc}\\ \sigma_{I,eq}^{0}=\sigma_{I,eq}f^{sc} \end{array}$$

where $f^{sc}$ is the scaling factor and listed in Table 3 for different failure modes. 

In order to alleviate the material softening behavior and stiffness degradation induced convergence difficulties, a viscous regularization method is needed for the numerical implementation. The viscous damage variable is expressed as:

(14) $${\dot{d}}_{I}^{v}=\frac{\left(d_{I}-d_{I}^{v}\right)}{\eta_{I}}$$

where $\eta_{I}$ denotes a viscous coefficient and $d_{I}^{v}$ is the regularized damage variable for each failure mode. By this method, the material tangent constitutive tensor is computed as:

(15) $$\frac{\partial \sigma }{\partial \varepsilon }=C_{d}+\varepsilon :\sum_{I}\frac{\partial C_{d}}{\partial d_{I}^{v}}\frac{\partial d_{I}^{v}}{\partial d_{I}}\frac{\partial d_{I}}{\partial f_{I}^{sc}}\frac{\partial f_{I}^{sc}}{\partial \varepsilon }$$

The aforementioned elastic-brittle damage behavior for the epoxy matrix was compiled as a user subroutine UMAT to realize the analysis.

It is well-known that the mismatch of thermal and chemical properties of constitutive materials at the micro-level combined with the layered and anisotropic nature of composite materials at the macro-level will lead to process-induced residual stresses in composite structures during the manufacturing process, which affect the mechanical properties and dimensional accuracy of the final product [34,35]. In other words, the samples were not at a stress free state before the external tension load was applied. So the simulations were divided into two steps taking the residual stresses into account. Firstly, the RVE model was subject to a homogeneous temperature reduction of 150 °C (from the processing temperature to room temperature and the processing temperature was assumed to be a stress free temperature) to represent the thermal residual stresses generated from the manufacturing process. Then, external tension load was applied by forced displacement of the nodes. The nodes in the *x* = *L* line were constrained to have an x-displacement of 0.02 mm. Because the length of the RVE is not so long, symmetric boundary conditions were applied in the *x* = 0 line. Meanwhile, symmetric boundary conditions were also applied in *y* = 0 line to reduce the computational cost. Four-node quadrilateral reduced integration plane strain element CPE4R and three-node triangle plane strain element CPE3 were used for the analysis where CPE4R elements were a majority. 

## 4. Results and Discussions

### 4.1. Experimental Results

#### 4.1.1. SEM Tests of the Cross Section of the Transverse Plies

**Figure 5:**
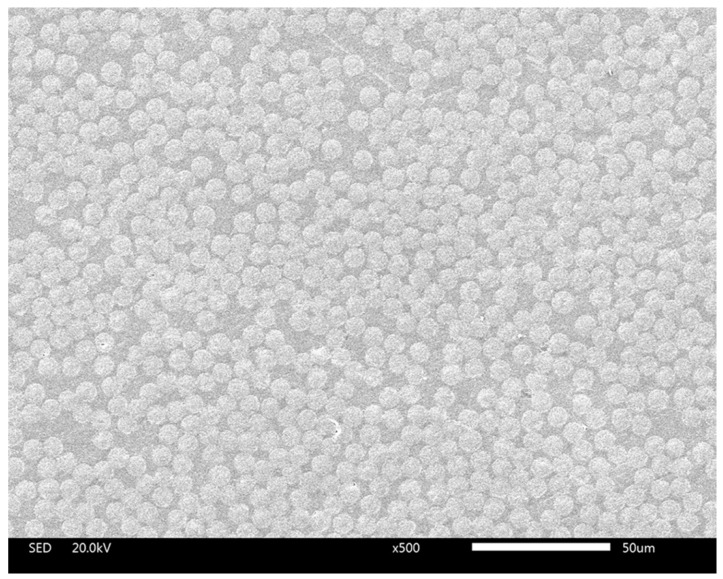
SEM results of the cross section of the transverse plies.

SEM results of the cross section of the transverse plies is shown in Figure 5. The fiber volume fraction was determined from Figure 5 by calculating the total area of the fibers through an imaging processing software ImageJ. The result of fiber volume fraction is 56.8% and the mean value of the radius of the AS4 carbon fiber is 3.9 mm. These two values were used to establish the microscopic transverse plies of the model in this paper.

#### 4.1.2. Uniaxial Tensile Test to Observe the Transverse Cracks

**Figure 6:**
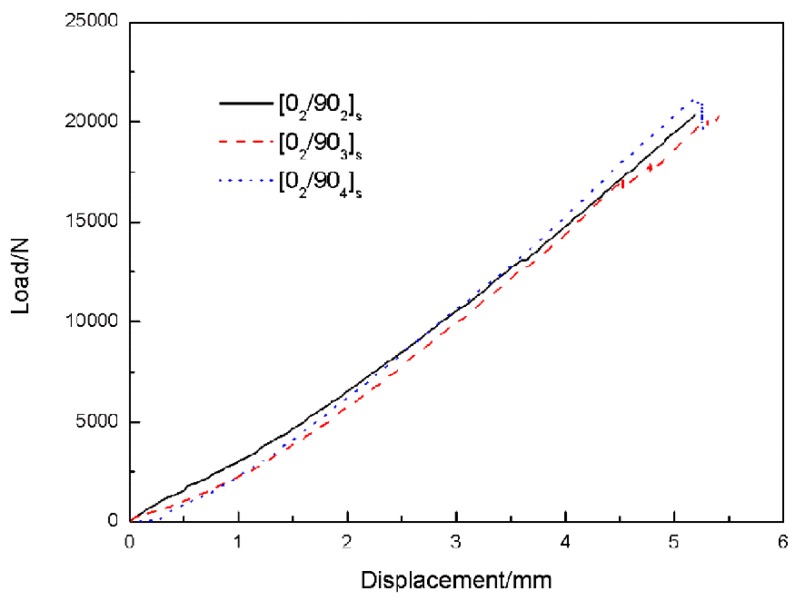
Typical load–displacement curves for the test samples.

Figure 6 gives the typical load–displacement curves for the test samples. In the early loading stage far from the final failure, slight fluctuations which may be caused by transverse matrix cracks could be observed. It should be noted that the peak loads of the samples showed insignificant differences. Because in comparison with the 90° layers, the loading capacity of the 0° layers is much larger and the number of the 0° layers in each laminate is identical.

**Figure 7:**
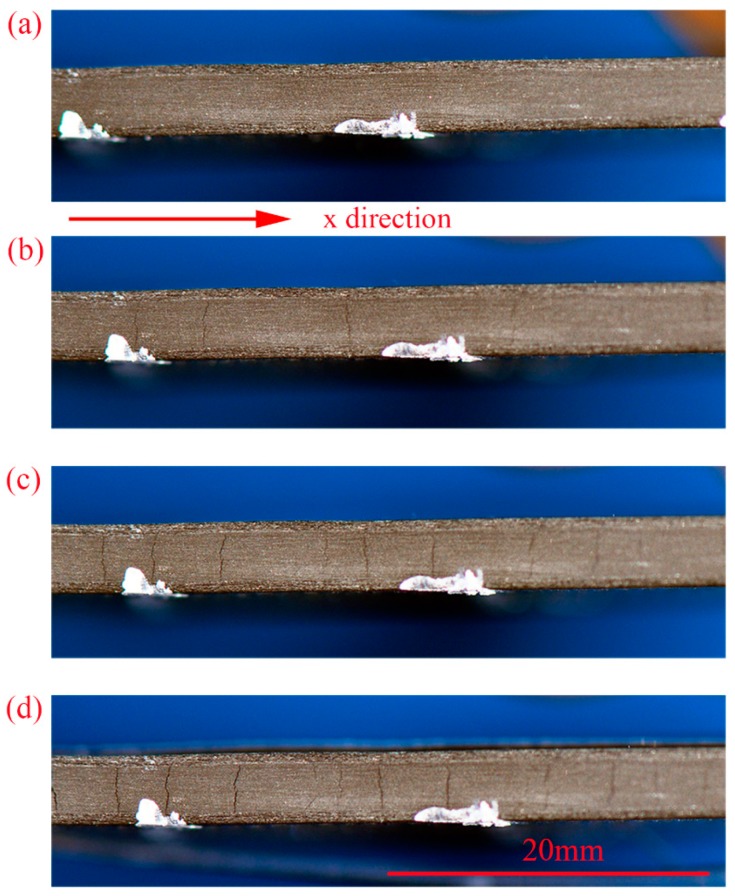
Photographs of transverse crack evolution of the [0_2_/90_4_]_s_ laminate at different times during the test captured after 6000 N: (**a**) 0 s; (**b**) 120 s; (**c**) 240 s; (**d**) 360 s.

**Figure 8:**
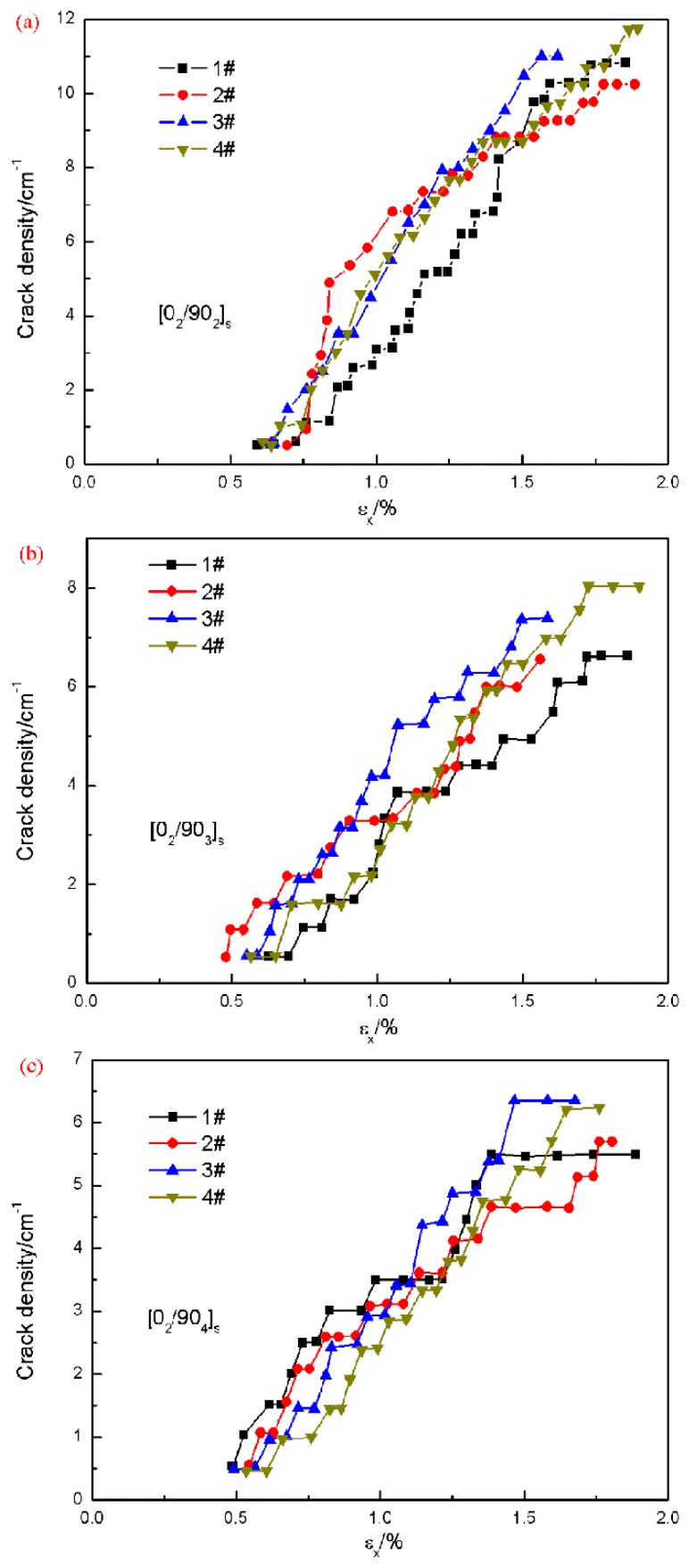
The experimentally observed crack density of the cross-ply [0_2_/90_n_]_s_ (n = 2,3,4) laminates under longitudinal uniaxial tension load. (**a**) [0_2_/90_2_]_s_ laminates; (**b**) [0_2_/90_3_]_s_ laminates; (**c**) [0_2_/90_4_]_s_ laminates.

Figure 7 illustrates the typical digital images captured after 6000 N for [0_2_/90_4_]_s_ laminate. The cracks can be easily observed with the naked eye. The cracks initiated at the transverse layers and had an intensive propagation. Then with the applied loads, multiple cracks continuously appeared until the number of cracks did not increase any more before the final failure, which means the saturated crack density was reached. The average saturation values of crack density are 10.9 /cm, 7.3 /cm and 5.9 /cm, respectively (see Figure 8) for laminates [0_2_/90_n_]_s_ of n = 2,3,4. Obviously, the crack spacing is lager in the laminates with thicker transverse layers. The developments of the transverse crack densities for all the cross-ply [0_2_/90_n_]_s_ (n = 2,3,4) laminates are given in Figure 8. It is shown that the cracks initiated at a critical load. The initial crack load for n = 2 is larger than the samples for n = 3 and the load for n = 3 is a little larger than the samples for n = 4. This indicates that the transverse layer thickness has an influence on the initial crack load. The increase of the thickness of the transverse layers leads to the decrease of the transverse strength. All of these results are consistent with what is reported in references [4,31].

### 4.2. Numerical Results

#### 4.2.1. Crack Initiation and Propagation

**Figure 9:**
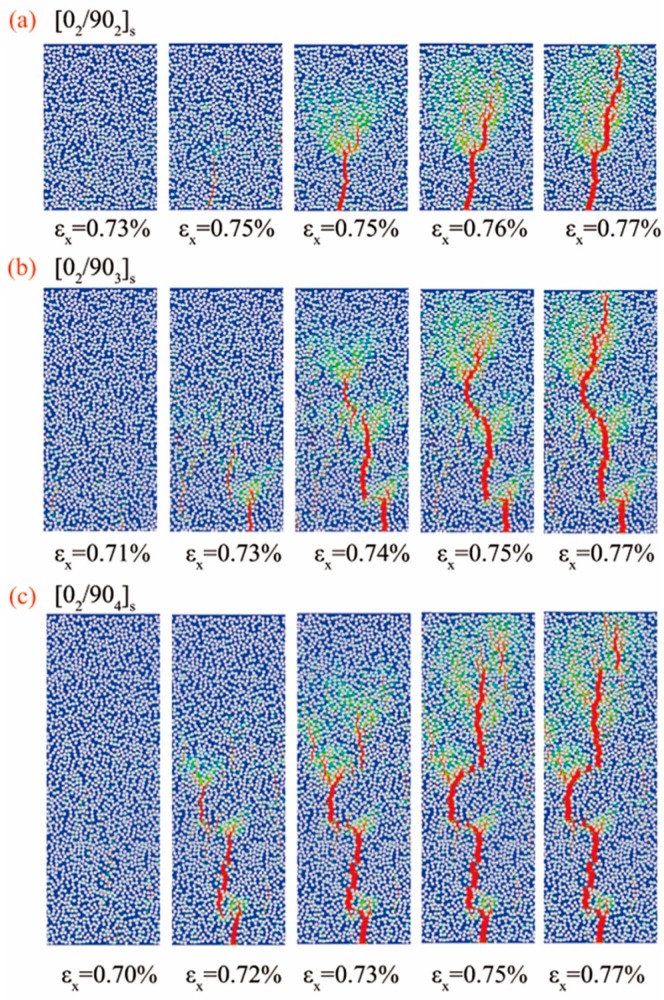
The simulation results of the development of the microcracks with the applied mechanical strain along *x* direction in the 90° plies of the cross-ply [0_2_/90_n_]_s_ (n = 2,3,4) laminate. (**a**) [0_2_/90_2_]_s_ laminate; (**b**) [0_2_/90_3_]_s_ laminate; (**c**) [0_2_/90_4_]_s_ laminate.

The development of the microcracks with the applied mechanical strain along *x* direction in the 90° plies of the cross-ply laminates is depicted in Figure 9. The onset and the propagation process of the microcracks are shown clearly. Damage started at the area between the fibers and then propagated very quickly along the adjacent domains to the 0° plies and was stopped at the interface between 0° and 90° plies. Many branches at the crack tip which guide the actual path of the crack were observed and one of them finally developed as the main path. This phenomenon is called as cracking branching behavior. Furthermore, the combination of the cracks was also observed. In Figure 9b,c, several initial cracks appeared in the early stages in different paths, with the load applied, the initial cracks combined and a main path of the crack was formed in the later stage. All the aforementioned phenomena were in good agreement with the experimental observations except for the crack initiation strains.

**Figure 10:**
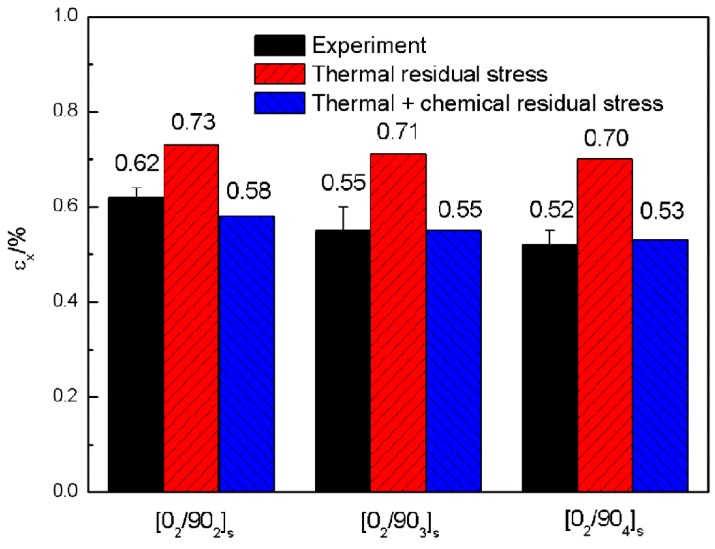
The relationship between the onset of the crack and the applied mechanical strain along *x* direction.

In the microscopic model, the crack initiates in the transverse layers could be captured accurately. The relationship between the onset of the crack and the applied mechanical strain $\varepsilon_{x}$ along x direction is shown in Figure 10. The onset of the crack corresponding to $\varepsilon_{x}$ was 0.62%, 0.55% and 0.52% for cross-ply [0_2_/90_n_]_s_ (n = 2,3,4) laminates in the experiment, respectively, and were 0.73%, 0.71% and 0.70% in the simulation, respectively. Apparently the simulation results were much higher than the experimental values. This is mainly due to the fact that the calculated residual stress was smaller than its true value. Thus, certain correction methods should be conducted. It is well known that thermal expansion and chemical shrinkage are the two main sources of process-induced residual stress. Only thermal residual stress is considered in the previous analysis. Thus, the computational stress was lower than its true value in the experiment under the same mechanical strain as a result of the absence of chemical shrinkage induced residual stress. 

**Figure 11:**
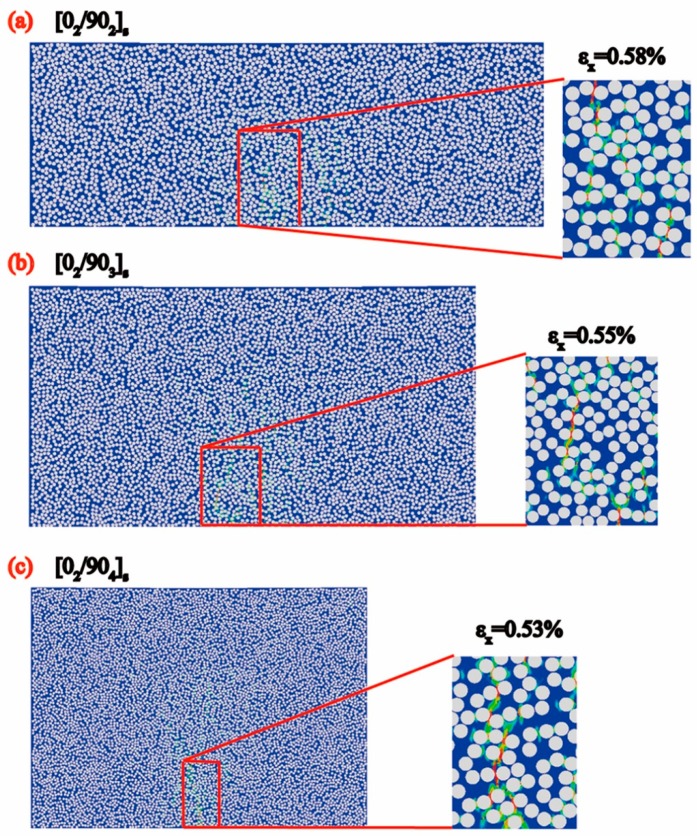
The simulation results of the initiation of the microcracks by the correction method of taking into account the thermal and chemical shrinkage residual stress with the applied mechanical strain along *x* direction in the 90° plies of the cross-ply [0_2_/90_n_]_s_ (n = 2,3,4) laminates. (**a**) [0_2_/90_2_]_s_ laminate; (**b**) [0_2_/90_3_]_s_ laminate; (**c**) [0_2_/90_4_]_s_ laminate.

According to the research of Ersoy et al. [36], the contribution of chemical shrinkage which leads to the spring-in phenomenon of the C-section cross-ply parts is between 31.4–57.9%. Assume that the contribution of chemical shrinkage in residual stress is 30% here and it follows the same rule with thermal residual stress. Therefore, the equivalent total residual stress could be computed by applying a temperature load of 214 °C temperature difference (stress free temperature has a 64 °C increase with adding in the contribution of chemical shrinkage). The recalculated results of the initiation of the microcracks, taking into account the thermal and chemical shrinkage residual stress with the applied strain $\varepsilon_{x}$ in the 90° plies of the cross-ply [0_2_/90_n_]_s_ (n = 2,3,4) laminates is shown in Figure 11. By taking into account the chemical shrinkage residual stress, the crack initiation strains were 0.58%, 0.55% and 0.53% for [0_2_/90_n_]_s_ (n = 2,3,4) laminates, respectively, which are very close to the experiment results. This demonstrates that crack initiation is very sensitive to residual stress. Larger residual stress will lead to earlier crack initiation in transverse plies. Therefore a high residual stress level induced by the manufacturing process is dangerous to composites. As the simulation result taking into account the equivalent total residual stress is closer to the experimental value, all the following discussions are under the frame of the total residual stress.

#### 4.2.2. In-Situ Transverse Ply Strength

The matrix strength of the sub layers in multidirectional laminates is apparently higher than that of unidirectional laminates with the same thickness and ply angles. This phenomenon is called as in-situ effect and the matrix strength is called as in-situ strength. Dvorak and Laws [4] give a relationship between the in situ transverse strength $Y_{T}^{tt}$ of thick plies to the transverse tensile strength $Y_{t}$ (equal to 81 MPa, supplied by the manufacturer [37]) as:

(16) $$Y_{T}^{tt}=1.12\sqrt{2}Y_{t}$$

**Figure 12:**
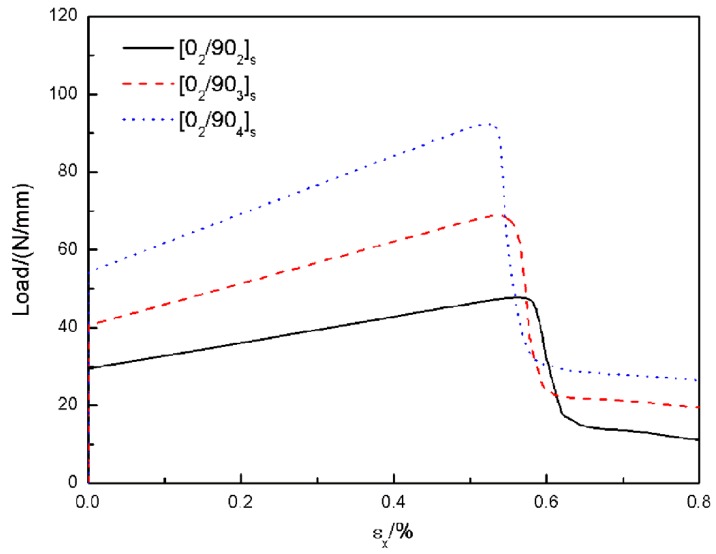
The load per unit width carried by the 90° plies of the three cross-ply laminates.

This in-situ transverse strength $Y_{T}^{tt}$ is actually the critical stress that leads to the through-thickness crack propagation. In the numerical model, the strength of the 90° plies, could be understood as the maximum stress carried by itself. Thus, according to solid mechanics, the stress of the 90° plies is easy to be calculated:

(17) $$\sigma_{x}=\frac{P_{tot}-P_{0}}{t_{90}}+\sigma_{x}^{r}$$

where $P_{tot}$ is the total force per unit width carried by the cross-ply laminate and $P_{0}$ is the force per unit width carried by the 0° plies respectively. Both of them can be obtained directly from the numerical results. Thus, the load carried by the 90° plies could be calculated. The results of load carried by the 90° plies of the three cross-ply laminates are shown in Figure 12. Obvious drops are observed in the three curves which indicates the degradation of load capacity. Therefore, the onset of each drop is where the crack initiates and it is consistent with the results discussed in Section 4.2.1. Here $t_{90}$ is the thickness for the 90° plies and $\sigma_{x}^{r}$ is the residual stress in the 90° plies and could be determined as the average stress carried by the 90° plies:

(18) $$\sigma_{x}^{r}=\frac{\sum_{i}\sigma_{x}^{ri}\Omega^{i}}{\sum_{i}\Omega^{i}}$$

where $\sigma_{x}^{ri}$ and $\Omega^{i}$ are the thermal residual stress along *x* direction and the area associated to the Gauss point *i* in the finite element discretization of the 90° plies, respectively. 

**Figure 13:**
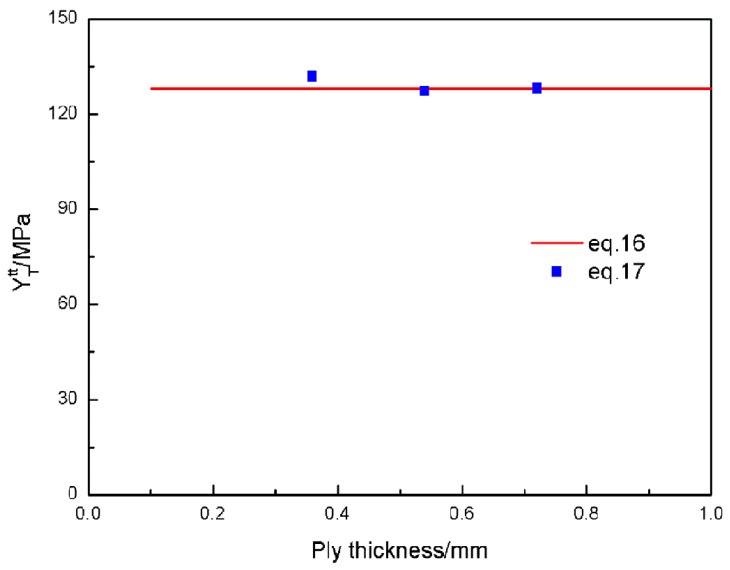
The relationship between the transverse ply strength and transverse ply thickness.

The results of the transverse ply strength are plotted as a function of ply thickness in Figure 13. The computational results of the transverse ply strength calculated by Equation (17) are very close to the results calculated by the empirical Equation (16). 

#### 4.2.3. Stiffness Degradation of the Transverse Ply

**Figure 14:**
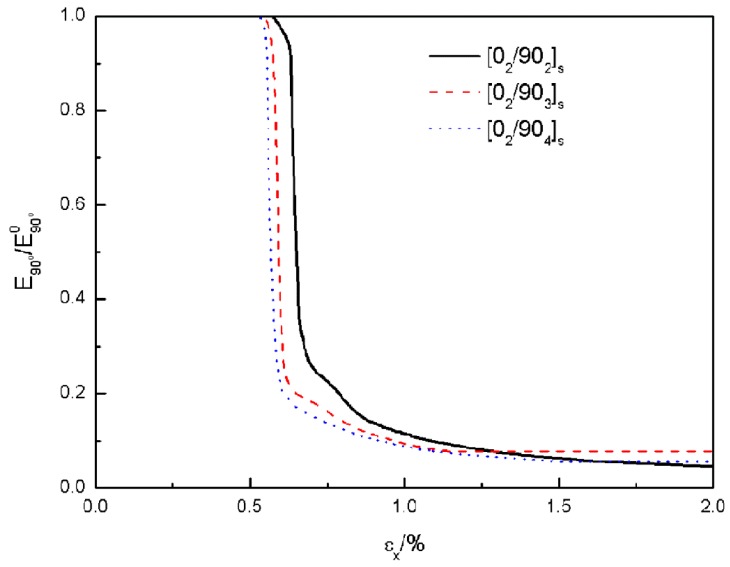
Predictions of the degradation of the normalized elastic modulus of the transverse ply.

Transverse cracks affect the load capacity of the transverse ply severely. Once the crack initiates, the stiffness of the transverse ply will degrade rapidly. The predictions of the degradation of the elastic modulus (normalized by the undamaged modulus) of the transverse ply is shown in Figure 14. The elastic modulus was computed as the average stress carried by the transverse ply along the *x* direction divided by the corresponding total strain (thermal strain and mechanical strain). The reduction of the modulus of the transverse ply was found a little earlier in thicker plies. But the magnitude of the declines were almost the same and showed a relationship independent of the transverse ply thickness. 

Although the loading case is macro uniaxial tension here, the proposed method is applicable for other loading cases like shear or tension–shear coupling, because the failure criteria contain tensile stress components and shear stress components. 

## 5. Conclusions

The transverse crack initiation and propagation behavior of cross-ply laminates under uniaxial tension load was studied by means of experimental and numerical methods. The experiment results show that the transverse crack initiated at critical loads and propagated rapidly to the interface between 0° and 90° plies. The transverse layer thickness has an influence on the initial crack load. 

In the numerical simulations, multi-scale RVEs were modeled to explore the transverse cracks in cross-ply [0_2_/90_n_]_s_ (n = 2,3,4) laminates. Only resin damage was considered in the analysis to avoid the severe convergence problems if fiber/matrix decohesion was considered. A second-order damage tensor was proposed to describe the damage of the resin. The damage initiation and evolution laws and the numerical implementation were discussed. It was found that the prediction values are consistent with the experiment results and the crack initiation is sensitive to residual stress. Evaluating the residual stress correctly is important for predicting the fracture behavior. The simulation results were very close to the experiment results by a correction method after the chemical shrinkage induced residual stress was considered. Both the experiment and simulation results show the onset of crack patterns with a lower longitudinal mechanical strain $\varepsilon_{x}$ in the laminates with thicker transverse plies. Furthermore, the prediction results of in-situ transverse ply strength and stiffness degradation of the transverse plies agree with the reference results well. 

By means of comparing the simulation results to experiment and reference results of the critical longitudinal mechanical strain $\varepsilon_{x}$ which initiates the cracks, the in-situ transverse ply strength and the stiffness degradation of the transverse plies, the simplified computational strategy proposed in this paper was proved valid to analyze transverse cracking of the cross-ply laminates.

## Figures and Tables

**Table 1:** Homogenized ply properties for AS4/8552 composites.

Longitudinal Young’s Modulus *E_1_*/GPa	Transverse Young’s Modulus *E_2_*/GPa	In-plane Poisson’s Ratio *ν_12_*	In-plane Shear Modulus *G_12_*/GPa	Longitudinal Coefficient of Thermal Expansion *α_1_*/(10^−6^K^−1^)	Transverse Coefficient of Thermal Expansion *α_2_*/(10^−6^K^−1^)
141	9.2	0.32	4.8	−0.34	34.4

**Table 2:** Mechanical properties for 8552 epoxy resin.

Modulus *E_m_*/GPa	Poisso-n’s Ratio *ν_m_*	Coefficient of Thermal Expansion *α*/(10^−6^K^−1^)	Tensile Strength *σ^t^_m_*/MPa	Compre-ssive Strength *σ^c^_m_*/MPa	Shear Strength *σ^s^_m_*/MPa	Critical Fracture Energy Release Rate *G_m_*/(J/m^2^)
5.1	0.35	52	121	176	50	90

**Table 3:** Equivalent displacement, equivalent stress and scaling functions in each failure mode.

Failure Mode	$\mathrm{Equivalent}\;\mathrm{Displacement}\;\delta_{eq}$	$\mathrm{Equivalent}\;\mathrm{Stress}\;\sigma_{eq}$	$\mathrm{Scaling}\;\mathrm{Function}\;f^{sc}$
**Tension in 1-drection** (${\hat{\sigma }}_{11}\geq 0$)	$L_{c}\sqrt{{\langle \varepsilon_{11}\rangle }^{2}+\varepsilon_{12}^{2}}$	$L_{c}\left(\langle \sigma_{11}\rangle \langle \varepsilon_{11}\rangle +\sigma_{12}\varepsilon_{12}\right)/\delta_{eq}^{1t}$	$1/\sqrt{F_{1t}}$
**Compression in 1-drection** (${\hat{\sigma }}_{11}<0$)	$L_{c}\sqrt{{\langle -\varepsilon_{11}\rangle }^{2}+\varepsilon_{12}^{2}}$	$L_{c}\left(\langle -\sigma_{11}\rangle \langle -\varepsilon_{11}\rangle +\sigma_{12}\varepsilon_{12}\right)/\delta_{eq}^{1c}$	$\frac{-\alpha_{1}+\sqrt{\alpha_{1}{}^{2}+4\beta_{1}}}{2\beta_{1}}$
**Tension in 2-drection** (${\hat{\sigma }}_{22}\geq 0$)	$L_{c}\sqrt{{\langle \varepsilon_{22}\rangle }^{2}+\varepsilon_{12}^{2}}$	$L_{c}\left(\langle \sigma_{22}\rangle \langle \varepsilon_{22}\rangle +\sigma_{12}\varepsilon_{12}\right)/\delta_{eq}^{2t}$	$1/\sqrt{F_{2t}}$
**Compression in 2-drection** (${\hat{\sigma }}_{22}<0$)	$L_{c}\sqrt{{\langle -\varepsilon_{22}\rangle }^{2}+\varepsilon_{12}^{2}}$	$L_{c}\left(\langle -\sigma_{22}\rangle \langle -\varepsilon_{22}\rangle +\sigma_{12}\varepsilon_{12}\right)/\delta_{eq}^{2c}$	$\frac{-\alpha_{2}+\sqrt{\alpha_{2}{}^{2}+4\beta_{2}}}{2\beta_{2}}$

Where $\alpha_{i}=\left[{\left(\frac{\sigma_{m}^{c}}{2\sigma_{m}^{s}}\right)}^{2}-1\right]\frac{{\hat{\sigma }}_{ii}}{\sigma_{m}^{c}}$, $\beta_{i}={\left(\frac{{\hat{\sigma }}_{ii}}{2\sigma_{m}^{s}}\right)}^{2}+{\left(\frac{{\hat{\sigma }}_{12}}{\sigma_{m}^{s}}\right)}^{2}$, i=1,2 and 〈 〉 is the Macauley bracket.
